# Supplementary material for: Patient-Derived PSEN1 Cerebral Organoids Revealed Parallel Development of Amyloid-β Accumulation and Network Dysfunction
Source: Cell Mol Neurobiol. 2026 Jul 20;46:152. doi: 10.1007/s10571-026-01779-7 (PMC13669279; doi:10.1007/s10571-026-01779-7)
Supplement: Supplementary file 1 [file 10571_2026_1779_MOESM1_ESM.pdf]

Supplemental Figure S1

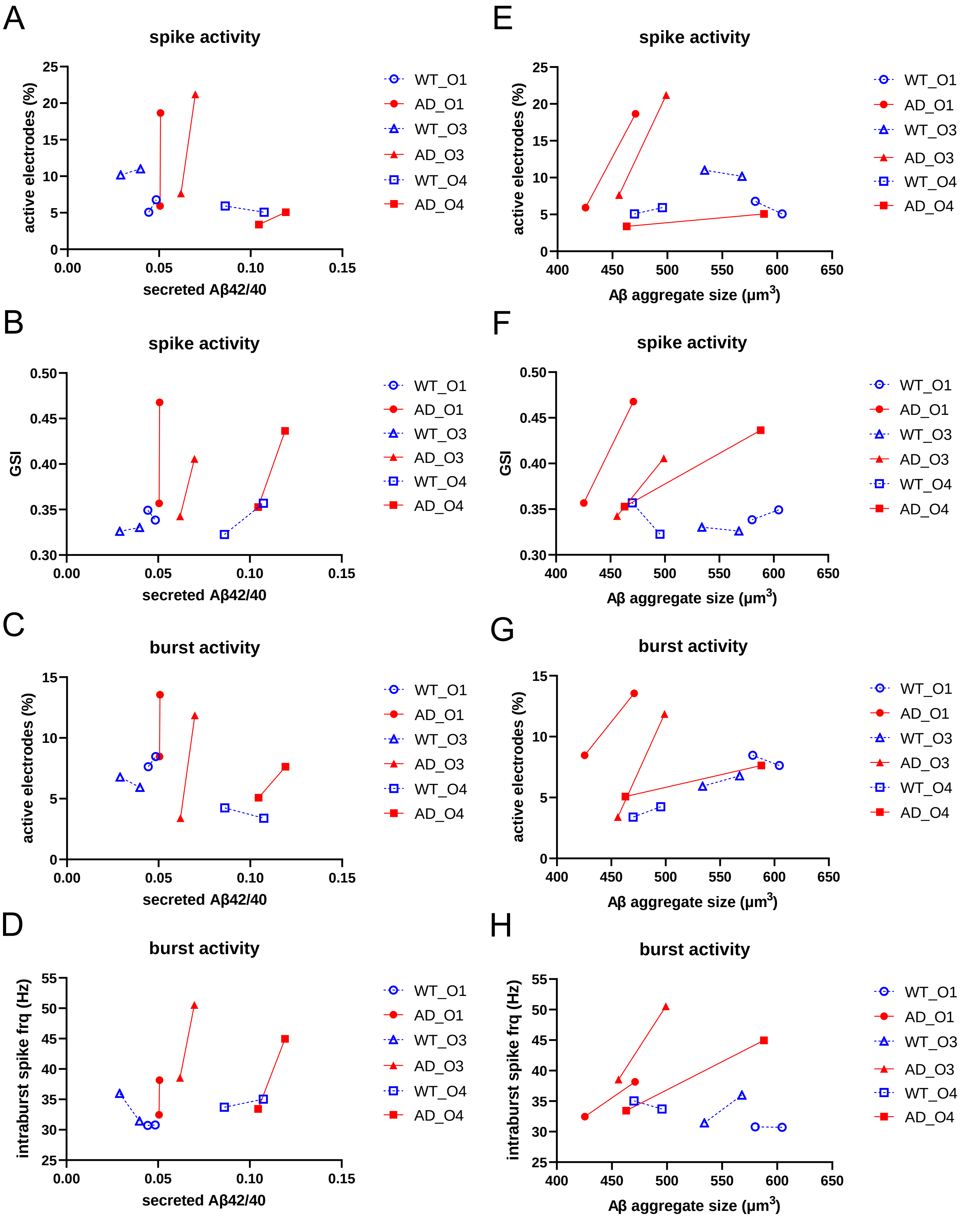

Supplementary Figure S1: Correlation between the electrical activity and Aβ in WT and AD cerebral organoids from three independent differentiations - data in absolute values.

A, B, C, D: Aβ42/40. **E, F, G, H:** Aβ aggregate size. Trends to a positive correlation can be mostly observed in AD (full red symbols), but not in WT (empty blue symbols), in both Aβ42/40 (A, B, C, D) and Aβ aggregate size (E, F, G, H).
