# Supplementary material for: Patient-Derived PSEN1 Cerebral Organoids Revealed Parallel Development of Amyloid-β Accumulation and Network Dysfunction
Source: Cell Mol Neurobiol. 2026 Jul 20;46:152. doi: 10.1007/s10571-026-01779-7 (PMC13669279; doi:10.1007/s10571-026-01779-7)
Supplement: Supplementary file 2 [file 10571_2026_1779_MOESM2_ESM.pdf]

Supplementary Table S1. Media, primers, and antibodies used in this study.

Cerebral Organoid Neural Induction Medium (NIM)

ITEM	COMPANY	IDENTIFIER	STORAGE CONCENTRATION	FINAL CONCENTRATION
DMEM/F12	Thermo Fisher Scientific	Cat# 21331046	Basal medium	Basal medium
N-2 Supplement	Thermo Fisher Scientific	Cat# 17502048	100X	1X
GlutaMAX™	Thermo Fisher Scientific	Cat# 35050061	100X	1X
MEM Non-Essential AA	Thermo Fisher Scientific	Cat# 11140035	100X	1X
Heparin sodium salt	Sigma	Cat# H3149	1 mg/mL (in H_2O)	1 µg/mL
2-Mercaptoethanol	Sigma	Cat# M3148	10mM (in PBS)	0.05mM
ZellShield®	Minerva Biolabs	Cat# 13-0050	100X	1X

Cerebral Organoid Differentiation Medium without vitamin A (CODM−A)

ITEM	COMPANY	IDENTIFIER	STORAGE CONCENTRATION	FINAL CONCENTRATION
DMEM/F12	Thermo Fisher Scientific	Cat# 21331046	Basal medium	1/2 of Basal medium
Neurobasal™	Thermo Fisher Scientific	Cat# 21103049	Basal medium	1/2 of Basal medium
N-2 Supplement	Thermo Fisher Scientific	Cat# 17502048	100X	0.5X
Insulin	Sigma	Cat# I9278	10 mg/mL	2.5 µg/mL
GlutaMAX™	Thermo Fisher Scientific	Cat# 35050061	100X	1X
MEM Non-Essential AA	Thermo Fisher Scientific	Cat# 11140035	100X	0.5X
B-27™ Supplement minus vitamin A	Thermo Fisher Scientific	Cat# 12587010	50X	0.5X
2-Mercaptoethanol	Sigma	Cat# M3148	10mM (in PBS)	0.1mM
ZellShield®	Minerva Biolabs	Cat# 13-0050	100X	1X

Cerebral Organoid Differentiation Medium with vitamin A (CODM+A)

ITEM	COMPANY	IDENTIFIER	STORAGE CONCENTRATION	FINAL CONCENTRATION
DMEM/F12	Thermo Fisher Scientific	Cat# 21331046	Basal medium	1/2 of Basal medium
Neurobasal™	Thermo Fisher Scientific	Cat# 21103049	Basal medium	1/2 of Basal medium
N-2 Supplement	Thermo Fisher Scientific	Cat# 17502048	100X	0.5X
Insulin	Sigma	Cat# I9278	10 mg/mL	2.5 µg/mL
GlutaMAX™	Thermo Fisher Scientific	Cat# 35050061	100X	1X
MEM Non-Essential AA	Thermo Fisher Scientific	Cat# 11140035	100X	0.5X
B-27™ Supplement	Thermo Fisher Scientific	Cat# 17504044	50X	0.5X
2-Mercaptoethanol	Sigma	Cat# M3148	10mM (in PBS)	0.1mM
ZellShield®	Minerva Biolabs	Cat# 13-0050	100X	1X

Cerebral Organoid MEA cultivation medium

ITEM	COMPANY	IDENTIFIER	STORAGE CONCENTRATION	FINAL CONCENTRATION
BrainPhys™ Neuronal Medium	STEMCELL Technologies	Cat# 05790	Basal medium	Basal medium
N-2 Supplement	Thermo Fisher Scientific	Cat# 17502048	100X	1X
B-27™ Supplement	Thermo Fisher Scientific	Cat# 17504044	50X	1X
ZellShield®	Minerva Biolabs	Cat# 13-0050	100X	0.5X

qPCR primers

TARGET	NCBI GENE ID	FW SEQUENCE 5’-3’	RV SEQUENCE 5’-3’
GAPDH	2597	AGCCACATCGCTCAGACAC	GCCCAATACGACCAAATCC
GFAP	2670	CCGACAGCAGGTCCATGT	GTTGCTGGACGCCATTG
MAP2	4133	TTGGTGCCGAGTGAGAAGA	GTCTGGCAGTGGTTGGTTAA
NEFM	4741	GAAATCGCTGCGTACAGAAAAC	TAATGGCTGTCAGGGCCTCTT
RBFOX3	146713	TACGCAGCCTACAGATACGCTC	TGGTTCCAATGCTGTAGGTCGC
SOX2	6657	TACAGCATGTCCTACTCGCAG	GAGGAAGAGGTAACCACAGGG
TUBB3	10381	TCAGCGTCTACTACAACGAGGC	GCCTGAAGAGATGTCCAAAGGC

Antibodies

ANTIBODY	COMPANY	IDENTIFIER	IHC DILUTION
β-Amyloid (D54D2)	Cell Signaling	Cat# 8243<br>RRID: AB_2797642	1:200
CTIP2	Cell Signaling	Cat# 12120<br>RRID: AB_2797823	1:200
NeuN	Millipore	Cat# MAB377<br>RRID: AB_2298772	1:200
MAP2	Millipore	Cat# AB5543<br>RRID: AB_571049	1:200
PAX6	Cell Signaling	Cat# 60433<br>RRID: AB_2797599	1:200
SOX2	Cell Signaling	Cat# 3579<br>RRID: AB_2195767	1:200
TUJ1	Aves Labs	Cat# TUJ-0020<br>RRID: AB_2313564	1:200
Hoechst 33342 Nucleic Acid Stain	Thermo Fisher Scientific	Cat# H3570<br>RRID: AB_3675235	1:1000
Donkey Anti-Chicken AF647	Jackson ImmunoResearch	Cat# 703-606-155<br>RRID: AB_2340380	1:400
Donkey Anti-Mouse AF488	Thermo Fisher Scientific	Cat# A-21202<br>RRID: AB_141607	1:400
Donkey Anti-Rabbit AF568	Thermo Fisher Scientific	Cat# A-10042<br>RRID: AB_2534017	1:400
Donkey Anti-Rabbit AF647	Thermo Fisher Scientific	Cat# A-31573<br>RRID: AB_2536183	1:400
